# Accurate temperature dependence of structure factors of l-alanine and taurine for quantum crystallography

**DOI:** 10.1107/S2052252525002647

**Published:** 2025-04-24

**Authors:** Mibuki Hayashi, Takashi Nishioka, Hidetaka Kasai, Eiji Nishibori

**Affiliations:** a Department of Physics, Institute of Pure and Applied Sciences and Tsukuba Research Center for Energy Materials Science University of Tsukuba Tsukuba305-8571 Japan; Warsaw University, Poland

**Keywords:** single-crystal diffraction data, l-alanine, taurine, quantum crystallography, twinning, X-ray constrained wavefunction, Hartree–Fock charge density

## Abstract

Multi-temperature high-quality structure factors of l-alanine and taurine were re-measured at the SPring-8 BL02B1 beamline for method development in quantum crystallography. The quality of the data was evaluated by comparison with previous studies. In the case of taurine, we found that the data quality was highly affected by small amounts of twinning. Residual electron density around the sulfur atoms observed in a previous study [Hibbs *et al.* (2003). *Chem. A Eur. J.***9**, 1075–1084] disappeared with the re-measured data. X-ray wavefunction refinements were carried out on these data. The difference electron density between the X-ray constrained wavefunction (XCW) results and the Hartree–Fock charge density showed a positive difference electron density around the nucleus and a negative difference electron density between the bonds. These features were consistent with those reported [Hupf *et al.* (2023). *J. Chem. Phys.***158**, 124103]. It was found that the deformation density around the nucleus and between bonds due to electron correlations and electronic polarization could be confirmed by the XCW method using the present structure factors.

## Abstract 2

The temperature dependence of accurate structure factors of l-alanine and taurine was measured at the SPring-8 BL02B1 beamline. The quality of the structure factors is evaluated by charge density and quantum crystallographic studies. The effects of small amounts of twinning on the charge density study for taurine are also described.

## 1. Introduction

Quantum crystallography (QCr) (Grabowsky *et al.*, 2017 ▸) studies based on accurate diffraction data have been carried out for decades (Koritsánszky & Coppens, 2001 ▸). The development of methods such as Hirshfeld atom refinement (HAR) (Capelli *et al.*, 2014 ▸) and the X-ray constrained wavefunction (XCW) method (Jayatilaka, 1998 ▸; Jayatilaka & Dittrich, 2008 ▸; Dittrich *et al.*, 2012 ▸; Grabowsky *et al.*, 2012 ▸) requires the most accurate experimental data. Such data have ultimately enabled the recent advances in QCr. One of the main goals of QCr is to improve theoretical computations based on quantum mechanics by accurate experimental measurements. Recent QCr studies have used accurate diffraction data to reveal the effects of electron correlation in chemical bonds, electron polarization in molecules (Genoni *et al.*, 2017 ▸; Hupf *et al.*, 2023 ▸) and van der Waals interactions (Kasai *et al.*, 2018 ▸) in materials. High-quality and high-resolution experimental structure factors have always been required for such developments and improvements of QCr methodology. There are a limited number of high-quality datasets available for method developments. Samples for method development are often limited to containing first- and second-row elements of the periodic table such as l-alanine and urea (Hupf *et al.*, 2023 ▸) at a single measurement temperature. The structure factors of l-alanine by Destro *et al.* (1988 ▸) have been extensively used for confirmation and method development in QCr. The effects of electron correlation in chemical bonds and electronic polarization of l-alanine in the crystal have been revealed by the XCW analysis (Hupf *et al.*, 2023 ▸). These data were also used for charge density studies by multipole modeling (MM) (Hansen & Coppens, 1978 ▸), HAR, molecule-in-cluster geometry optimization and restraint validation (Dittrich *et al.*, 2020 ▸), and normal mode refinement (Hoser & Madsen, 2016 ▸). Taurine, a sulfur-containing zwitterion, has recently attracted attention due to its anti-aging effects (Singh *et al.*, 2023 ▸). Hibbs *et al.* (2003 ▸) reported the MM charge density of taurine (Hibbs *et al.*, 2003 ▸). They found that the largest residual electron density was in the region close to the sulfur atom. This residual density remained even after QCr MOON refinement, which is a molecular orbital occupation number refinement (Waller *et al.*, 2006 ▸).

Changing the temperature is known to affect the structure as well as the charge density. Increasing temperature smears (Hirshfeld, 1976 ▸) the charge density of materials. Therefore, QCr studies are usually carried out using low-temperature diffraction data measured at less than 100 K to reduce thermal effects. Recent progress in dynamical QCr to treat phonon dispersion can, in principle, quantitatively evaluate thermal motion in crystallography (Hoser & Madsen, 2016 ▸). However, there are very few good-quality charge-density datasets available that have been measured at different temperatures for dynamical QCr.

Advances in X-ray sources, detectors as well as software allow us to routinely measure diffraction data of a quality suitable for hydrogen position determination by HAR and chemical bonding studies of normal σ bonds using state-of-the-art laboratory diffractometers. However, to reveal small effects such as electron correlation in chemical bonds, highly accurate diffraction data with sufficient counting statistics and high resolution in reciprocal space with *d* > 0.3 Å resolution are required. Over the past 15 years, we have developed and perfected measurement techniques for accurate high-resolution single-crystal data with high reciprocal resolution at the SPring-8 BL02B1 beamline (Sugimoto *et al.*, 2010 ▸). The quality of the charge density study of CoSb_3_ (skutterudite) using the previous imaging plate detector has been reported, comparing diffraction data measured at other facilities and with other diffractometers (Schmøkel *et al.*, 2013 ▸). Other examples include the high-quality charge density studies of TiS_2_ (Kasai *et al.*, 2018 ▸), of a Dy-containing single molecular magnet (Gao *et al.*, 2020 ▸) and a Ti-containing Mott insulator (Kitou *et al.*, 2020 ▸). Since then, a CdTe PILATUS 3X detector has been installed at the BL02B1 beamline. The properties of such detectors have been carefully investigated by charge density studies (Krause *et al.*, 2020 ▸) and several high-quality charge density studies have already been reported using this detector type (Kitou *et al.*, 2023 ▸; Vosegaard *et al.*, 2022 ▸). In the present study, multi-temperature synchrotron single-crystal X-ray diffraction data of l-alanine and taurine were re-measured at the SPring-8 BL02B1 beamline with the CdTe detector. Data quality was evaluated using MM, HAR and XCW methods. In addition, theoretical structure factors of l-alanine and taurine were calculated using the program *CRYSTAL14* (Dovesi *et al.*, 2014 ▸). Theoretical structure factors were also analyzed by the XCW method for comparison.

## 2. Experiment and analysis

### 2.1. Sample preparation

Commercially available small crystals of l-alanine and taurine were purchased. Small crystals of l-alanine and taurine less than 200 µm in the longest dimension were selected and mounted on the top of a glass fiber with an ep­oxy resin. The quality of the sample was estimated by the measurement of single-crystal data using an XtaLAB mini at the University of Tsukuba. Several pre-selected samples were measured at the SPring-8 BL02B1 beamline. The best-quality sample was selected using the above procedures. The criteria of the sample quality were evaluated using the shape of the Bragg peaks in the high-angle region, the intensities of Bragg peaks, and the results of indexing and data reduction.

### 2.2. Multi-temperature synchrotron radiation single-crystal experiments

Multi-temperature synchrotron radiation single-crystal X-ray diffraction experiments were carried out at the SPring-8 BL02B1 beamline. The incident X-ray energy was 50 keV. The temperature of the samples was controlled by N_2_ and He gas-flow low-temperature devices. The temperature was calibrated using a thermocouple at the sample position before measurements. The temperature fluctuation was ±0.5 K. Preliminary measurements of 180° ω scans with fine slicing were performed for each sample to determine an optimal exposure time for the multi-temperature data. The exposure time of one frame for the pre-experiment was 0.1 s. The exposure times of the multi-temperature data for l-alanine and taurine were determined as 1.0 and 0.8 s from the pre-experiments. The data collection at each temperature typically consisted of 10800 fine-sliced frame images with two 2θ angle and three χ angle settings. The data collection for l-alanine at 100 K consisted of 18000 fine-sliced frame images with two 2θ angles and three χ angles addition to two ϕ angle settings. Using these preliminary measurements, we were able to avoid nonlinearity problems (Krause *et al.*, 2020 ▸) in our multi-temperature data. The taurine diffraction data were measured two times since small amounts of twinning were detected in the data of the first measurement, which will be described later.

### 2.3. Data reduction and further analysis

The data were processed with the program *CrysAlisPro* (Rigaku, 2022 ▸). All tif files output from the PILATUS detector were converted to Esperanto format by *CrysAlisPro*. Then, peak hunting, unit-cell finding and data reduction were carried out for each temperature of data collection. Three datasets with the high 2θ angle setting for taurine were not used in the analysis due to the weak intensities of the diffraction peaks. The extracted data were merged using the *SORTAV* software (Blessing, 1997 ▸). Initial structure analysis was performed with the *SHELX* suite (Sheldrick, 2008 ▸) and the graphical user interface of the *Olex2* system (Dolomanov *et al.*, 2009 ▸). The space group, initial atomic coordinates and anisotropic displacement parameters were determined in the process. Diffraction data were analyzed by MM, HAR and the XCW method using *XD2016* (Volkov *et al.*, 2016 ▸), *ORCA* (Neese, 2012 ▸) with *Olex2*, and *Tonto* (Jayatilaka & Grimwood, 2003 ▸). The local coordinate system of atoms in the l-alanine and taurine molecules for MM used connecting atoms inside the molecules. The local atomic site symmetries of atoms in the molecules were determined from the molecular structure. Local symmetries of all hydrogen atoms were set to cylinder symmetry. HAR refinements were carried out using the *NoSpherA2* option (Kleemiss *et al.*, 2021 ▸) in *Olex2*. The program *ORCA* was used for the update table, which is a calculation of atomic scattering factors for bonded atoms. The basis set was 6-31G(d) and the density functional BLYP. The conditions for HAR were zero total charge of a molecule, multiplicity of the wavefunction was one and the self-consistent field (SCF) strategy for normal convergence. In the XCW method, the 6-31G(d) basis set was again used in the analysis. The analysis was performed using both Hartree–Fock (HF) and density functional theory (DFT). The λ in the analysis was increased from 0.0 with 0.05 steps. The analysis was continued until the SCF cycle was unable to converge. The *CRYSTAL14* program (Dovesi *et al.*, 2014 ▸) was used to perform a single-point energy calculation with the functional/basis-set representation B3LYP/POB-TZVP-rev2 for the experimental geometry. The experimental lattice constants were not optimized in the calculation. To smooth convergence, a level-shifting value of 0.6 Hartree was set for the molecular orbitals of the Fock matrix. Theoretical structure factors were calculated by the program *CRYSTAL14* for comparison with the experimental data.

## 3. Results and discussion

### 3.1. Refinement results of IAM, HAR, MM and XCW analysis for multi-temperature data

Initial structure analysis was carried out using the program *SHELXL* (Sheldrick, 2008 ▸). Table 1 ▸ shows a list of refinement statistics of the temperature-dependence data for l-alanine and taurine. All datasets have an average redundancy of more than 10. The completeness of all the datasets exceeded 96.5% with a reciprocal resolution of 1.67 Å^−1^. *R*_1_ and *wR*_2_ were as small as 0.037 and 0.092. There was a *checkCIF* B-alert on the rigid-bond test for all the taurine data.

HAR was carried out for the temperature-dependent datasets using DFT with the B3LYP functional and the 6-31G(d) basis set. Table 2 ▸ shows the refinement statistics of HAR for l-alanine and taurine. *R*_1_ and *wR*_2_ for HAR were improved by 0.003 to 0.008 and 0.001 to 0.017, respectively, compared with those from the *SHELX* refinement for l-alanine. *R*_1_ and *wR*_2_ for HAR were improved by 0.004 to 0.005 and 0.02 to 0.02, respectively, compared with those of the *SHELX* refinement for taurine. The B-alert of *checkCIF* for taurine data at 150 K disappeared.

MM refinements were also carried out for these data. Anharmonic thermal parameters up to the fourth order were added for C, N, O and S atoms. Table 3 ▸ lists *R*, *R*_w_ and goodness of fit (GooF) for the MM refinements. The improvement in *R* and *R*_w_ was again significant. *R* is around 1% for l-alanine and around 2% for taurine. Overall *R* for the MM improved by more than 0.015 compared with the *SHELX* refinement for l-alanine and taurine.

Next, XCW analyses were carried out for the temperature-dependent diffraction data. Four kinds of analyses were performed. XCW analysis, which is a combination of four calculation steps, was carried out for each sample and each data point. The XCW method involves fitting a wavefunction to the X-ray diffraction data, which helps to accurately reconstruct the charge density within a crystal. A single-molecule wavefunction and a cluster around the central molecule were compared. The cluster around the central molecule for each symmetry-generated molecule within a radius of 8 Å was used for the analysis. Table 4 ▸ lists *R*_1_, *wR*_2_ and GooF for the XCW analysis of l-alanine using a cluster arrangement and DFT. The B3LYP functional and *POB-TZVP-rev2* (Kleemiss *et al.*, 2021 ▸) basis set were used for the DFT calculation. Values for *R*_1_ span from 0.0206 to 0.0318. *wR*_2_ values were in the range 0.0195 to 0.1028.

**Figure 1:**
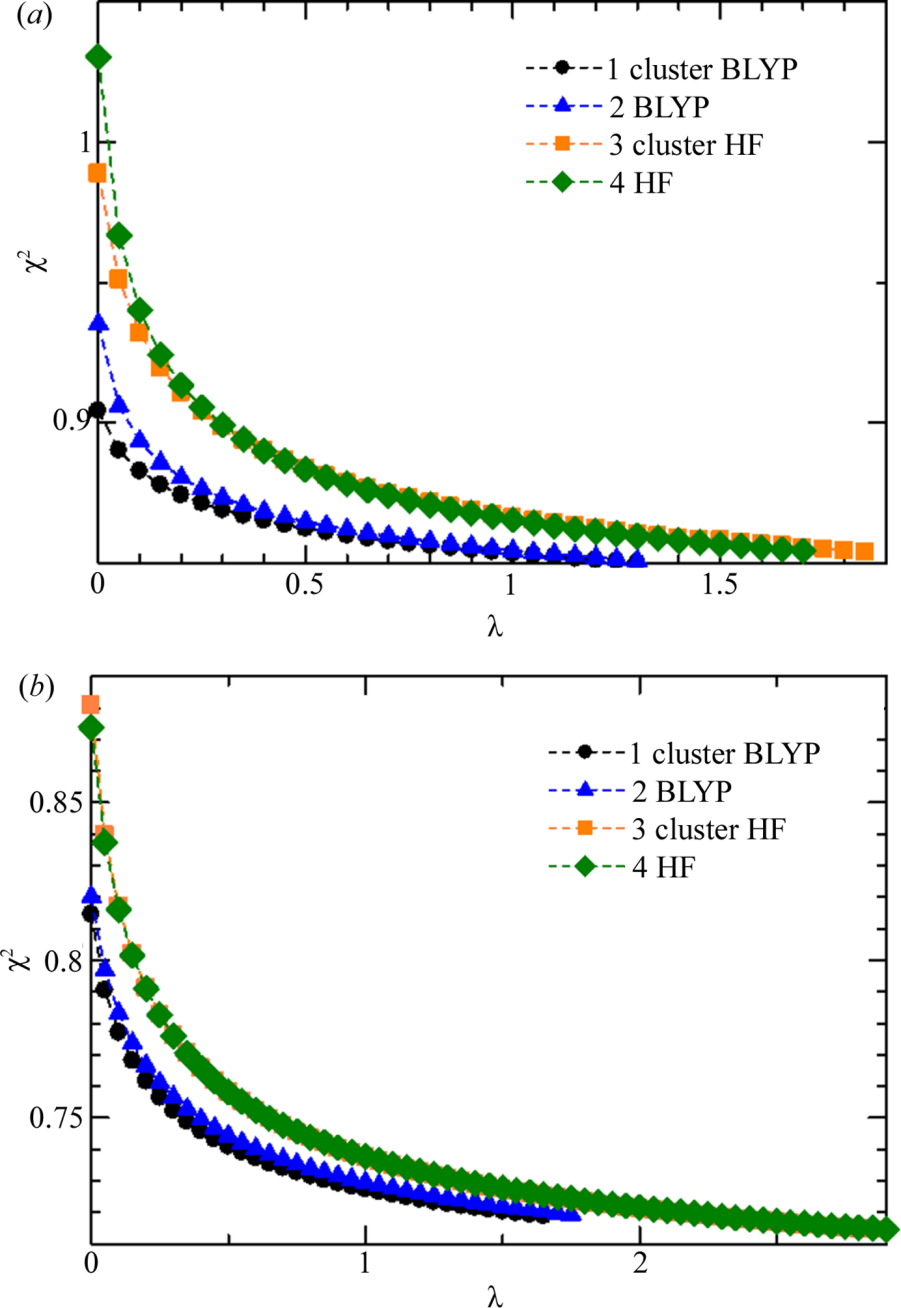
λ dependence of χ^2^ for the XCR analysis: (*a*) l-alanine at 40 K, (*b*) taurine at 85 K.

Fig. 1 ▸ shows the λ dependence of χ^2^ for the four types of XCW analyses. Fig. 1 ▸(*a*) shows l-alanine at 40 K and Fig. 1 ▸(*b*) is taurine at 85 K. It can be recognized that the most realistic analysis condition, which is a combination of a cluster model and DFT as indicated by black circles, gave the lowest χ^2^ at λ = 0.0 for both l-alanine and taurine. The facts indicate that the present experimental data include detailed information on the charge density such as electron correlation and electron polarization which are not included in a single-molecule HF calculation. χ^2^ drastically decreased from λ = 0.0 to λ = 0.5. Then, χ^2^ gradually decreased with increasing λ.

### 3.2. Comparison with the previous data

So far, accurate single-crystal diffraction data of l-alanine and taurine were reported by Destro *et al.* (1988 ▸) and Hibbs *et al.* (2003 ▸) using laboratory X-ray sources and diffractometers. The l-alanine data of Destro *et al.* (1988 ▸) were widely used for QCr studies including HAR, XCW, NoMoRe and MM methods (Destro *et al.*, 1988 ▸). The taurine data of Hibbs were analyzed by MM refinement. We compared the refinements of the present datasets with these previous analyses. The reciprocal resolution of the 23 K data of Destro *et al.* (1988 ▸) was (sin θ)/λ_max_ = 1.0778 Å^−1^, which was lower than that of any of the present temperature-dependence data. *R*_1_ of the IAM refinement of the previous data was 0.0320, which was much higher than that of the present dataset. *R*_1_ of the IAM refinement of the present 40 K data was 0.0273 with (sin θ)/λ_max_ = 1.6667 Å^−1^ reciprocal resolution. *R*_1_ of MM of the previous data was 0.0203 which was much higher than that of the previous 40 K data, 0.0113, with (sin θ)/λ_max_ = 1.6667 Å^−1^ resolution. *R*_1_ of HAR for the previous data was 0.019. *R*_1_ of HAR for the present data at 40 K with (sin θ)/λ_max_ = 1.6667 Å^−1^ reciprocal resolution was 0.026. *R*_1_ of the present full-resolution data was higher than that of the previous data. *R*_1_ of the present data with the same resolution of the previous data, (sin θ)/λ_max_ = 1.0778 Å^−1^, was 0.0119. The quality of the present data was at least comparable and probably even better than that of the previous data in the refinement.

**Figure 2:**
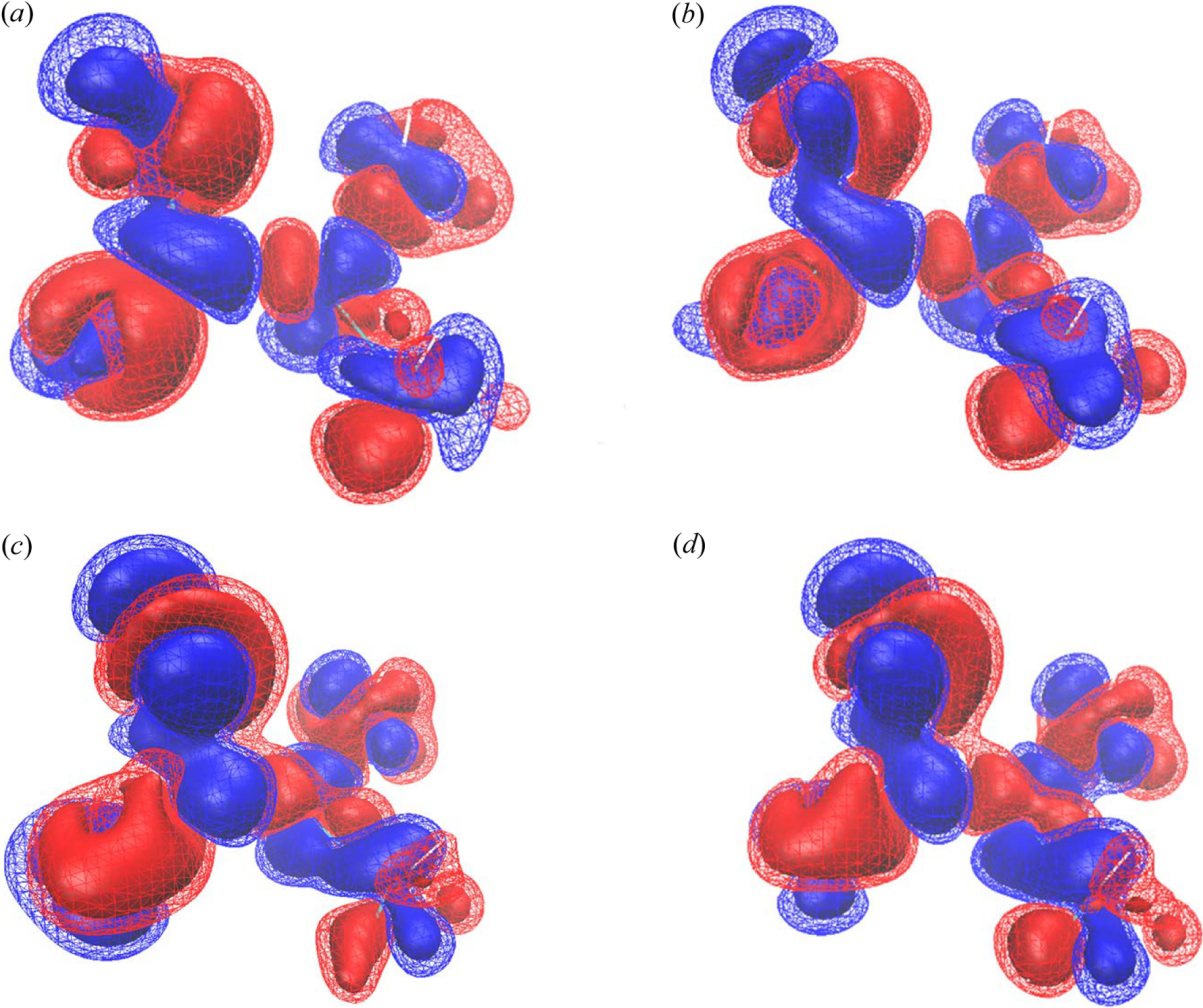
Deformation density due to the electron correlation and polarization determined from the present data. (*a*) 40 K, (*b*) 100 K, (*c*) 150 K and (*d*) 200 K. The red solid and mesh surfaces are −0.005 and −0.0025 a.u. The blue solid and mesh surfaces are 0.005 and 0.0025 a.u.

Fig. 2 ▸ shows the deformation density due to the electron correlation and the electron polarization determined from the present data. The deformation density was calculated as the difference electron density between the XCW result and a single-molecule HF calculation. Figs. 2 ▸(*a*)–2 ▸(*e*) are 40, 100, 150 and 200 K, in order. The difference density between λ = λ_max_ to λ = 0.0 by a single-molecule model with HF calculation is shown in the figure to show the correlation and polarization. The red solid and mesh surfaces are −0.005 and −0.0025 a.u. The blue solid and mesh surfaces are 0.005 and 0.0025 a.u. At all temperatures, a blue positive electron density was observed around the nucleus, and a red negative differential electron density was observed between bonds. This is consistent with the study by Hupf *et al.* (2023 ▸). The data measured in this study can be extracted from the effects of electronic correlations and polarization is contained in the experimental data in small amounts.

In the present 85 K data of taurine, (sin θ)/λ_max_ = 1.3736 Å^−1^ with *R*_1_ = 0.0235 and *wR*_2_ = 0.0723. The number of measured reflections was 147423 and the number of independent reflections was 10090. The taurine 100 K data from Hibbs *et al.* (2003 ▸) were (sin θ)/λ_max_ = 1.240 Å^−1^ with *R*_1_ = 0.023 and *wR*_2_ = 0.058. *R*_1_ and *wR*_2_ of the dataset at 85 K performed at (sin θ)/λ_max_ = 1.240 Å^−1^ – which was the same resolution as the data of Hibbs *et al.* (2003 ▸) – were 0.0205 and 0.0647. *R*_1_ of the present 85 K data was 0.0025 lower than the data from Hibbs *et al.* (2003 ▸) and *wR*_2_ was 0.0067 higher. The present data had a lower *R*_1_ than the Hibbs *et al.* (2003 ▸) data, even though the number of measured reflections was more than 6 times greater. The completeness was also 100%, which was better than the 97% of Hibbs *et al.* (2003 ▸).

MM refinement for the present 85 K data with (sin θ)/λ_max_ = 1.3736 Å^−1^ resulted in *R*_1_ = 0.0128 and *R*_w_ = 0.0259. The refinement with (sin θ)/λ_max_ = 1.240 Å^−1^ for comparison with the data of Hibbs *et al.* (2003 ▸) resulted in *R*_1_ = 0.0128 and *R*_w_ = 0.0261. In the MM of taurine at 100 K in Hibbs *et al.* (2003 ▸), *R*_1_ = 0.018 and *R*_w_ = 0.035.

#### 3.2.1. Findings of small amounts of twinning from the accurate analysis for taurine

In the present study, we found that charge density refinement can indicate of small amounts of twinning. Twinning affected the residual electron density distribution and can be detected by MM refinement and QCr studies of taurine. We believe that the taurine crystal studied in Sections 2 and 3 was not affected by twinning. We also performed MM analysis on our own twinned sample data in the present study. In order to distinguish the no-twinning sample, the sample in which twinning is found is called taurine-2. At 85 K, MM refinement of no-twinning taurine resulted in *R*_1_ = 0.0113, *R*_w_ = 0.0246 and GooF = 0.9478. At 40 K, MM refinement of taurine-2 resulted in *R*_1_ = 0.0184, *R*_w_ = 0.0358 and GooF = 2.2319. The GooF of taurine-2 was 1.2841 greater than that of taurine. In the difference electron density map after MM refinement of taurine-2, a difference electron density was observed around the sulfur atom that was not seen in the no-twinning sample.

**Figure 3:**
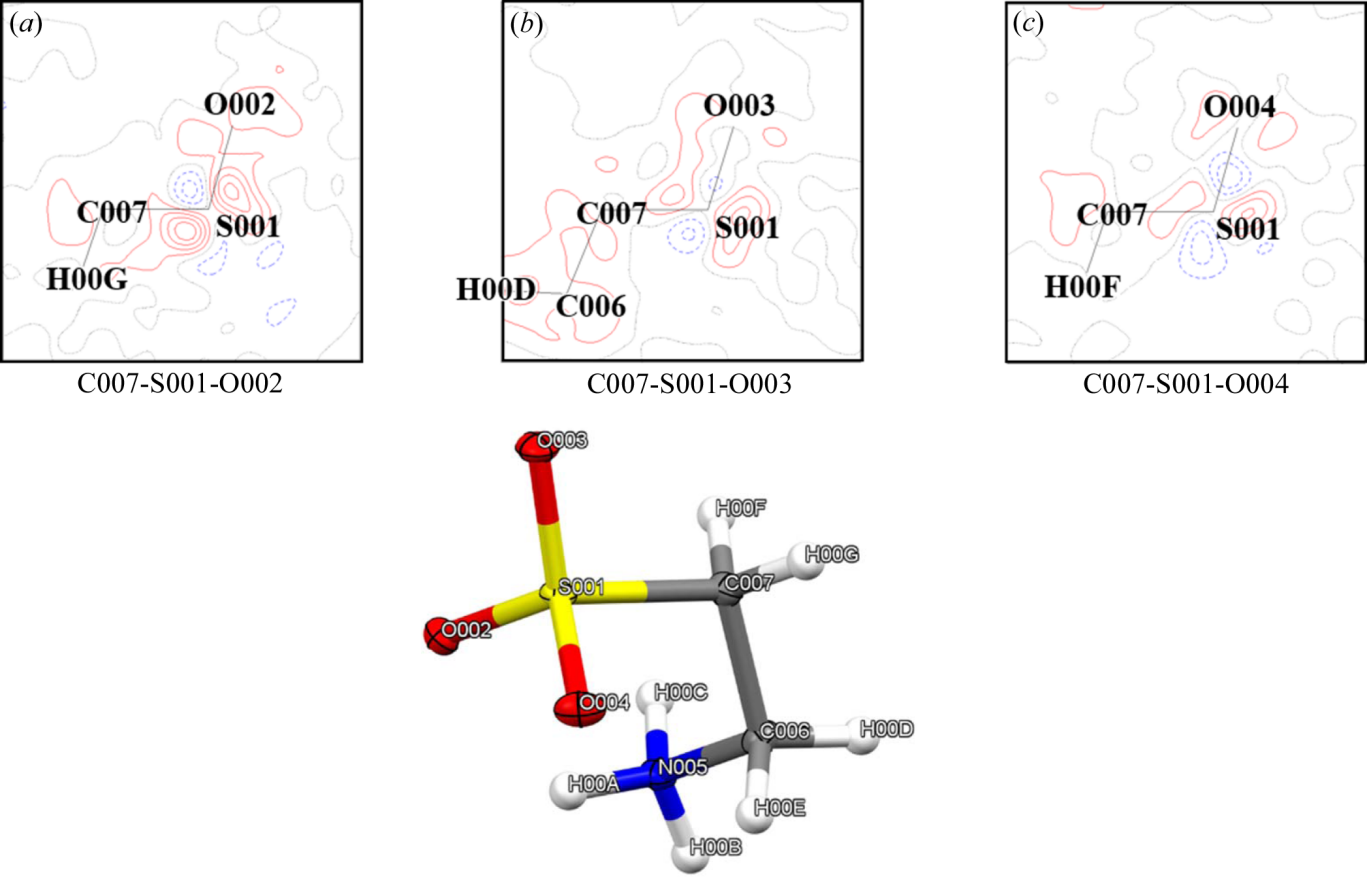
Difference electron density maps of taurine-2 at 40 K with the molecular structure. (*a*) C007–S001–O002, (*b*) C007–S001–O003 and (*c*) C007–S001–O004 atoms were on the plane. The contour lines were drawn at the 0.1 e Å^−3^ step. Red lines are positive, bule dashed lines are negative and the black solid line is 0.0 e Å^−3^.

Fig. 3 ▸ shows a difference electron density map of taurine-2 at 40 K with its molecular structure. This map is the difference between the experimental data and the electron density refined by MM. The difference electron density between the bonds disappeared after MM refinement. Difference electron densities were observed around the sulfur atoms similar to the previous studies by Hibbs *et al.* (2003 ▸). We consider this a strong indicator for the presence of minor twinning in the earlier study.

**Figure 4:**
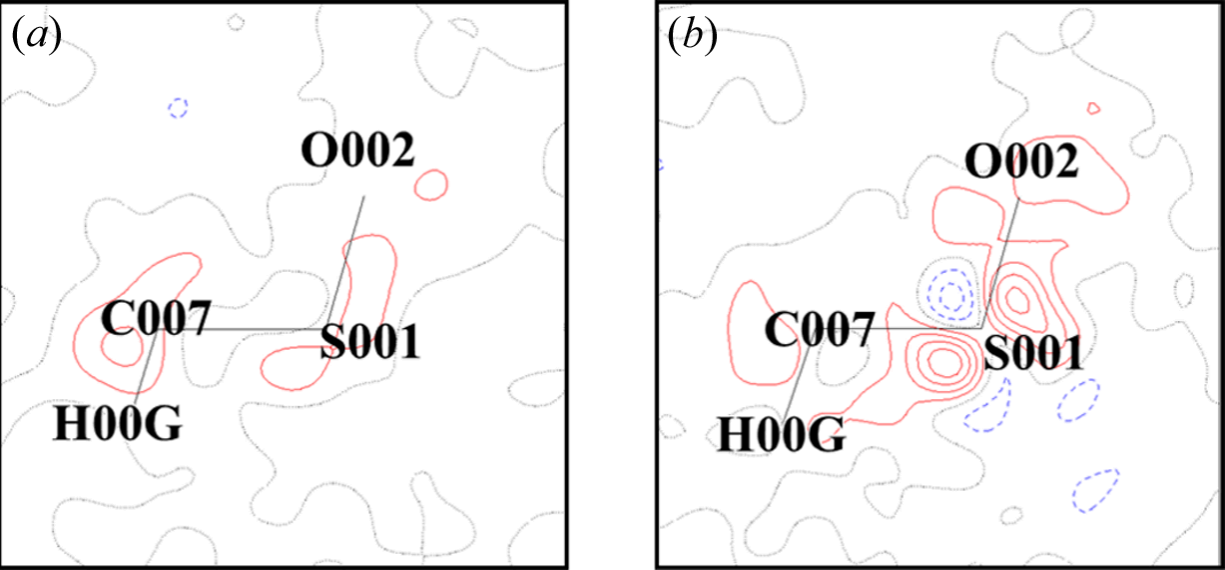
Difference electron density map of the C007–S001–O002 plane in the MM refinement of (*a*) the no-twinning sample and (*b*) taurine-2. The levels of the lines are the same as those in Fig. 3 ▸.

Fig. 4 ▸ shows a difference electron density map of the C007–S001–O002 plane in the MM refinement of the no-twinning sample and taurine-2. The no-twinning sample had no difference electron densities around sulfur atoms. Data from taurine-2 showed electron density difference around the sulfur atoms similar to the previous study by Hibbs *et al.* (2003 ▸).

Table 5 ▸ shows the ratio of indexed reflections for the no-twinning sample and taurine-2. From the left, the columns show the name of the measurement dataset, the number of indexed reflections, the number of unindexed reflections and the percentage of indexed reflections. The percentages of indexed reflections of the no-twinning sample and taurine-2 were 99.03 and 96.77%, respectively.

Taurine-2 was indexed as a twin crystal. There was a possibility that reflections that did not appear in the diffraction of a single crystal would appear when a twinned sample was investigated. This could be seen from the fact that the ratio of indexed reflections of taurine-2, which had the difference electron density around the sulfur atom, was 96.77%, which was 2.26% lower than that of the no-twinning sample. Two types of components were used in the twin indexing. There were 79764 reflections of component 1, 1707 reflections of component 2, 1389 overlapped reflections and 989 unindexed reflections. The reflections of components 1 and 2 were 96.8 and 3.7%, respectively. Overall, the proportion of reflections with integer exponents was 98.82%.

QCr revealed that difference electron density appeared around the sulfur atom of taurine due to minor twinning. Table 5 ▸ shows that the ratio of indexed reflections of taurine-2 is 96.77%. *R*_1_ and *R*_w_ after MM refinement of taurine-2 were 0.0184 and 0.0358, respectively. The *R*_1_ and *R*_w_ values after MM refinement in Hibbs *et al.* (2003 ▸) were 0.018 and 0.035, respectively. The data for taurine-2 were sufficiently low in *R*_1_ and *R*_w_ compared with the previous data. The ratio of indexed reflections with the non-twinned sample, which was considered to be a single crystal, was 99.03%. This ratio was 2.26% higher than that of taurine-2. As shown in Fig. 4 ▸, there was no difference electron density between carbon–carbon bonds. Therefore, we conclude that strong bonds such as carbon–carbon are sufficiently well reproduced from the data even with a small amount of twinning. A slight twinning effect is manifested in the difference electron density around the sulfur atom by performing MM refinement.

### 3.3. Temperature dependence of XCW analysis for taurine

In the present study, we extracted the effects of electron correlations in chemical bonds and electronic polarization of taurine from XCW analysis using diffraction data in the absence of twinning. The analysis of structure factors from *CRYSTAL14* can serve as a theoretical reference. First, the XCW analysis of *CRYSTAL14* structure factors was described. Then, the XCW analysis of experimental data was shown in comparison with *CRYSTAL14* results.

**Figure 5:**
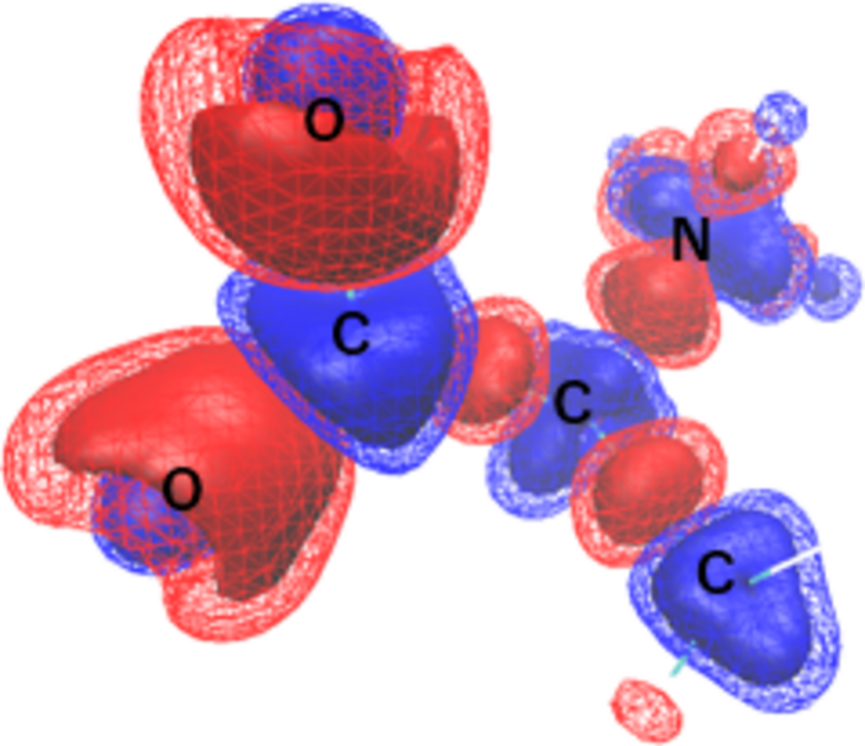
Deformation of the electron density due to the electron correlation extracted by the XCW method using the *CRYSTAL* structure factors of l-alanine. The electron density surface level shown is the same as in Fig. 2 ▸.

XCW analysis of the theoretical structure factor from *CRYSTAL14* for l-alanine was additionally performed to evaluate the quality of the structure factors. The deformation density due to electron correlation extracted by XCW analysis of l-alanine was comparable to the work of Hupf *et al.* (2023 ▸). To confirm the quality of the structure factors from *CRYSTAL14*, XCW analysis using these structure factors was also performed for l-alanine under the same conditions as for taurine. The SCF calculation of the *CRYSTAL* calculation of l-alanine was completed in 13 cycles. The total energy was −1294.8 a.u. with this method/basis-set selection. Fig. 5 ▸ shows the deformation of the electron density due to the electron correlation extracted by XCW analysis using the *CRYSTAL* structure factors of l-alanine. The figure shows a difference electron density between λ = 0.0 (using HF theory) and XCW fitted λ = 5.0. This difference electron density represented the deformation of the electron density due to electron correlation as extracted by the XCW method. The surface level of the electron density shown was the same as in Fig. 2 ▸. A positive difference electron density was observed around the nucleus, and a negative difference electron density was observed between covalent bonds. These features were almost consistent with those reported by Hupf *et al.* (2023 ▸). It was found that the deformation density around the nucleus and between bonds due to electron correlations and electronic polarization could be reconstructed by the XCW method using the *CRYSTAL* structure factors.

**Figure 6:**
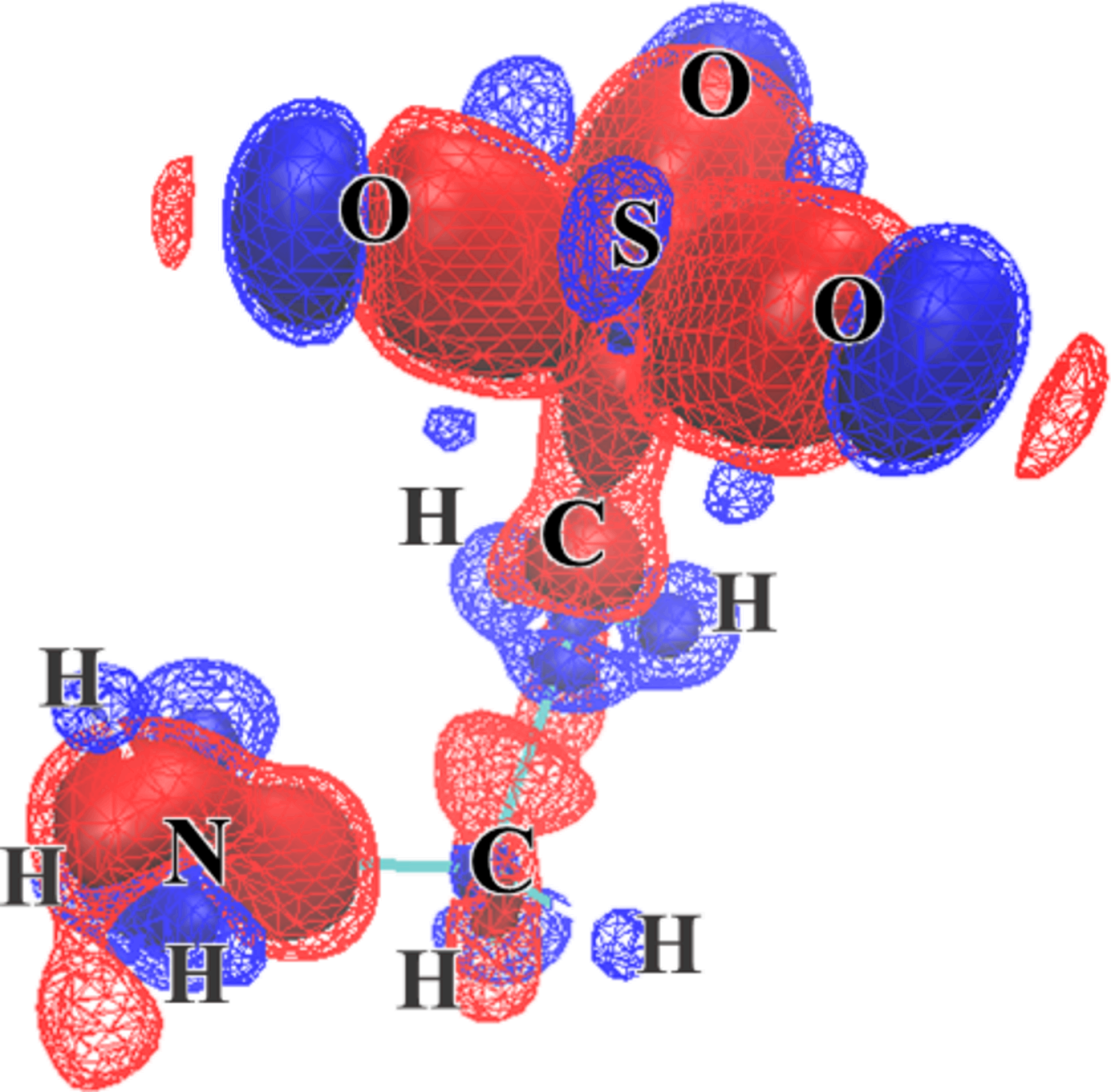
Deformation of the electron density due to the electron correlation extracted by the XCW method using the *CRYSTAL* structure factors of taurine. The electron density surface level shown is the same as in Fig. 2 ▸.

The SCF calculation for taurine used the same method and basis set. It was completed after 11 cycles, and the total energy was −3035.3 a.u. The calculated structure factors were again used for XCW analysis. Fig. 6 ▸ shows the deformation density due to the electron correlation extracted by the XCW method using the *CRYSTAL14* structure factors of taurine. Difference electron density between λ = 0.0 and λ_max_ is shown. The red solid and mesh surfaces are −0.0025 and −0.00125 a.u. The blue solid and mesh surfaces are 0.0025 and 0.00125 a.u. As with l-alanine, positive difference electron densities were observed around the nucleus and negative difference electron densities were observed between covalent bonds. The negative difference between the bonds of the oxygen and sulfur atoms spread around the oxygen atoms.

**Figure 7:**
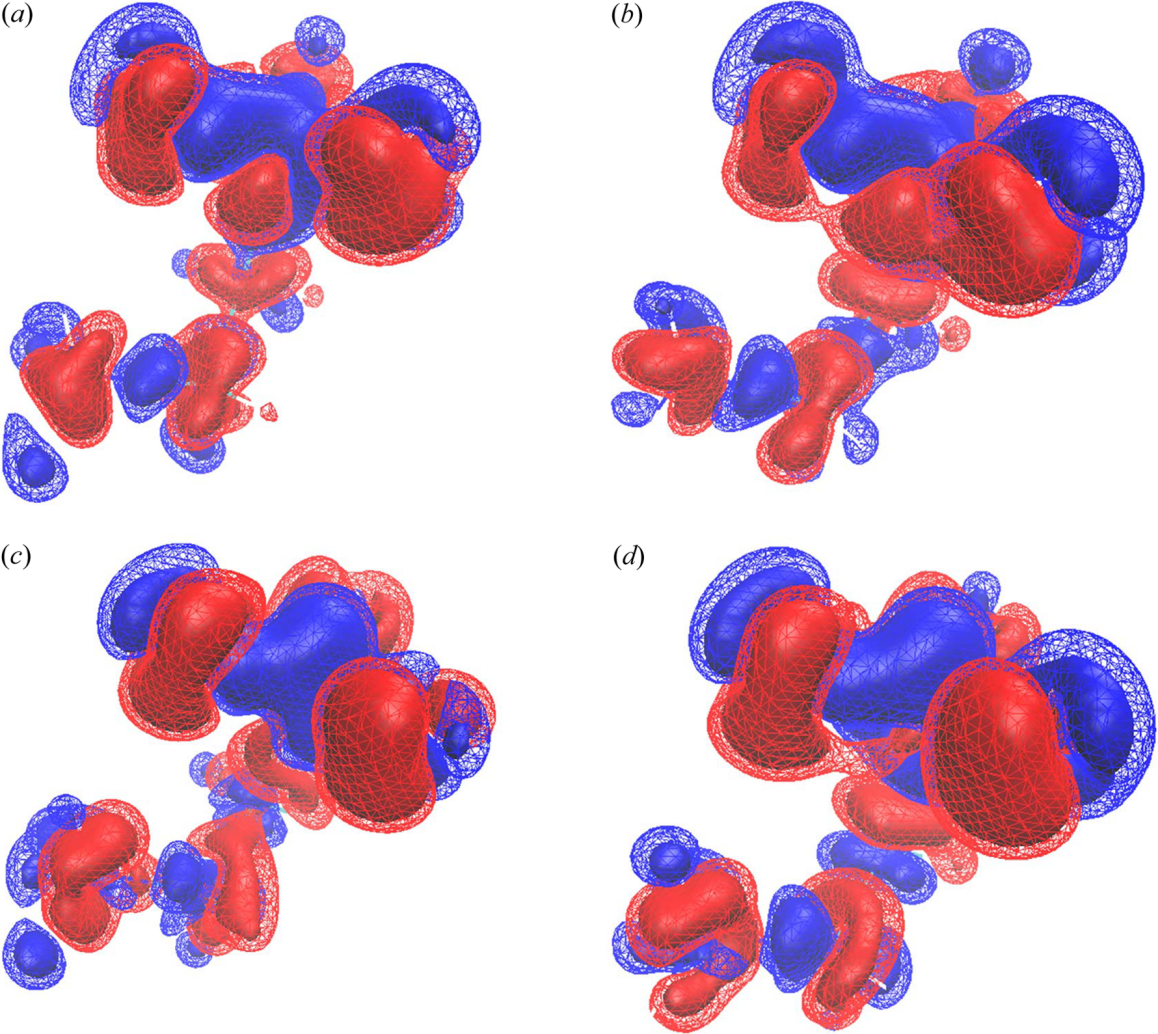
Difference electron density using the XCW method for the temperature dependence of the taurine data. The electron density surface level shown is the same as in Fig. 2 ▸.

Fig. 7 ▸ shows the difference electron density between λ = 0.0 and λ_max_ for the XCW method using taurine data at 85 K. Fig. 7 ▸(*a*) is the BLYP with cluster calculation, Fig. 7 ▸(*b*) is the BLYP without cluster calculation, Fig. 7 ▸(*c*) is the HF with cluster calculation and Fig. 7 ▸(*d*) is the HF without cluster calculation. The electron density surface level shown is the same as in Fig. 6 ▸. In the XCW analysis of taurine, effects that could not be expressed by the theory excluding the polarization and electron correlation of nitro­gen and carbon atoms of Fig. 7 ▸(*a*), and the deformation of the electron density due to the polarization of Fig. 7 ▸(*d*), resulted in a negative electron density around the nucleus, and a positive difference between bonds.

This was consistent with the XCW results for the taurine *CRYSTAL14* structure factors shown in Fig. 6 ▸. The positive difference electron density around the sulfur atom of taurine for Figs. 7 ▸(*c*) and 7 ▸(*d*) was wider than that for Figs. 7 ▸(*a*) and 7 ▸(*b*). This was qualitatively similar to the positive difference electron density around the sulfur atom that was not seen in Fig. 6 ▸.

## 4. Conclusions

In this study, we measured and evaluated the quality of high-quality temperature-dependence diffraction data of l-alanine and taurine. By evaluating three types of analyses, namely MM, HAR and XCW, it was found that the measured temperature-dependence data can be used productively for QCr research. Using synchrotron radiation and data with a high signal-to-noise ratio, it was possible to identify a very small number of factors that contribute to reducing data quality, such as slight twinning in the example of taurine as reported earlier. In the future, we believe the data measured in this study will be used to advance the methodology of QCr. New techniques of QCr, which include tools such as periodic HAR, have been developed recently. We hope that the present data will be used for the further development of these approaches.

## Supplementary Material

Crystal structure: contains datablock(s) lalanine8_40k, lalanine8_100k_run10, lalanine8_150k, lalanine8_200k, taurine51_85k, taurine51_150k, taurine51_200k. DOI: 10.1107/S2052252525002647/woz5001sup1.cif

Supporting tables. DOI: 10.1107/S2052252525002647/woz5001sup2.pdf

Unmerged hkl values for L-alanine 40K. DOI: 10.1107/S2052252525002647/woz5001sup3.txt

Unmerged hkl values for L-alanine 100K. DOI: 10.1107/S2052252525002647/woz5001sup4.txt

Unmerged hkl values for L-alanine 150K. DOI: 10.1107/S2052252525002647/woz5001sup5.txt

Unmerged hkl values for L-alanine 200K. DOI: 10.1107/S2052252525002647/woz5001sup6.txt

Unmerged hkl values for taurine 85K. DOI: 10.1107/S2052252525002647/woz5001sup7.txt

Unmerged hkl values for taurine 150K. DOI: 10.1107/S2052252525002647/woz5001sup8.txt

Unmerged hkl values for taurine 200K. DOI: 10.1107/S2052252525002647/woz5001sup9.txt

Sum file of L-alanine 40K. DOI: 10.1107/S2052252525002647/woz5001sup10.txt

Sum file of L-alanine 100K. DOI: 10.1107/S2052252525002647/woz5001sup11.txt

Sum file of L-alanine 150K. DOI: 10.1107/S2052252525002647/woz5001sup12.txt

Sum file of L-alanine 200K. DOI: 10.1107/S2052252525002647/woz5001sup13.txt

Sum file of taurine 85K. DOI: 10.1107/S2052252525002647/woz5001sup14.txt

Sum file of taurine 150K. DOI: 10.1107/S2052252525002647/woz5001sup15.txt

Sum file of taurine 200K. DOI: 10.1107/S2052252525002647/woz5001sup16.txt

CCDC references: 2443206, 2443207, 2443208, 2443209, 2443210, 2443211, 2443212

## Figures and Tables

**Table 1:** Refinement statistics for the independent-atom model (IAM) for temperature-dependence data for L-alanine and taurine

Sample	Temperature (K)	(sin θ)/λ_max_ (Å^−1^)	*R*_1_	*wR*_2_	*R*_int_	Average redundancy	Completeness (%)
L-Alanine	40	1.6667	0.0273	0.0655	0.0718	10.4	96.6
	100	1.4706	0.0240	0.0638	0.0739	20.4	99.9
	150	1.3514	0.0310	0.0798	0.0762	14.2	99.9
	200	1.2821	0.0366	0.0914	0.0776	15.4	100.0
Taurine	85	1.3736	0.0235	0.0723	0.0756	13.9	100.0
	150	1.1900	0.0287	0.0786	0.0831	10.1	100.0
	200	1.1110	0.0302	0.0852	0.0836	10.0	100.0

**Table 2:** Refinement statistics of HAR for temperature-dependence data for L-alanine and taurine

Sample	Temperature (K)	(sin θ)/λ_max_ (Å^−1^)	*R*_1_	*wR*_2_	GooF
L-Alanine	40	1.6667	0.0260	0.0639	0.8680
	100	1.4706	0.0194	0.0456	1.0605
	150	1.3514	0.0244	0.0634	0.7882
	200	1.2821	0.0286	0.0729	0.8575
Taurine	85	1.3736	0.0197	0.0510	1.0147
	150	1.190	0.0242	0.0567	1.0061
	200	1.111	0.0252	0.0591	1.0422

**Table 3:** Refinement statistics of MM for temperature-dependence data for L-alanine and taurine

Sample	Temperature (K)	(sin θ)/λ_max_ (Å^−1^)	*R*	*R*_w_	GooF
L-Alanine	40	1.6667	0.0103	0.0233	0.8727
	100	1.4706	0.0081	0.0221	0.7790
	150	1.3514	0.0097	0.0225	0.7996
	200	1.2821	0.0095	0.0222	0.7907
Taurine	85	1.3736	0.0122	0.0250	1.0197
	150	1.190	0.0179	0.0379	0.9813
	200	1.111	0.0175	0.0369	0.9441

**Table 4:** Refinement statistics for the XCW method for temperature-dependence data of L-alanine and taurine

Sample	Temperature (K)	(sin θ)/λ_max_ (Å^−1^)	λ_max_	*R*_1_	*wR*_2_	GooF
L-Alanine	40	1.6667	1.30	0.0273	0.0525	0.9220
	100	1.4706	0.85	0.0208	0.0196	0.9505
	150	1.3514	1.30	0.0275	0.0861	1.0139
	200	1.2821	0.65	0.0318	0.0550	1.1386
Taurine	85	1.3736	1.65	0.0248	0.0525	0.8478
	150	1.190	2.15	0.0315	0.0923	0.9151
	200	1.111	2.35	0.0327	0.0617	0.9371

**Table 5:** Ratio of indexed reflections of the no-twinning sample and taurine-2

Data	Indexed reflections	Unindexed reflections	Ratio of indexed reflections (%)
No-twinning sample	13801	135	99.03
Taurine-2	81141	2708	96.77
Taurine-2 (twinning)	Component 1: 79764	1389	Component 1: 96.8
	Component 2: 1707		Component 2: 3.7
