# Supplementary material for: Accurate temperature dependence of structure factors of l-alanine and taurine for quantum crystallography
Source: IUCrJ. 2025 Apr 24;12(Pt 3):384–92. doi: 10.1107/S2052252525002647 (PMC12044860; doi:10.1107/S2052252525002647)
Supplement: Supplementary file 2 [file m-12-00384-sup2.pdf]

IUCrJ

Volume 12 (2025)

Supporting information for article:

Accurate temperature dependence of structure factors of L-alanine and taurine for Quantum Crystallography

Mibuki Hayashi, Takashi Nishioka, Hidetaka Kasai and Eiji Nishibori

Link https://doi.org/10.5281/zenodo.14688662

Raw data sets of temperature dependent of L-alanine and taurine data by Esperanto format.

Table S1 Local symmetries of atoms for L-alanine

Atom	Local axis 1	Vector for local axis 1	Local axis 2	Vector for local symmetry	Local symmetry
O001	X	C004	Y	O002	m
O002	X	C004	Y	O001	m
N003	Z	C005	X	H00A	3m
C004	Z	C005	X	O001	mm2
C005	X	N003	Y	C004	no
C006	Z	C005	X	H00D	3m
H00A	Z	N003	Y	C005	cyliner
H00B	Z	N003	Y	C005	cylinder
H00C	Z	N003	Y	C005	cylinder
H005	Z	C005	Y	N003	cylinder
H00D	Z	C006	Y	C005	cylinder
H00E	Z	C006	Y	C005	cylinder
H00F	Z	C006	Y	C005	cylinder

Table S2 Refined MM parameters for L-alanine

Atom	Monopole	Dipole	Quadrupole	Octupole	Hexadecapole
O001	M1	D1+, D1-	Q0, Q2+, Q2-	O1+, O1-, O3+, O3-	H0, H2+, H2-, H4+, H4-
O002	M1	D1+, D1-	Q0, Q2+, Q2-	O1+, O1-, O3+, O3-	H0, H2+, H2-, H4+, H4-
N003	M1	D0	Q0	O0, O3+	H0, H3+
C004	M1	D0	Q0, Q2+	O0, 2+	H0, H2+, H4+
C005	M1	D0, D1+, D1-	Q0, Q1+, Q1-, Q2+, Q2-	O0, O1+, O1-, O2+, O2-, O3+, O3-	H0, H1+, H1-, H2+, H2-, H3+, H3-, H4+, H4-
C006	M1	D0	Q0	O0, O3+	H0, H3+
H00A	M1	D0			
H00B	M1	D0			
H00C	M1	D0			
H005	M1	D0			
H00D	M1	D0			
H00E	M1	D0			
H00F	M1	D0			

Table S3 Dipole parameters of L-alanine at 40K.

	D11+	D11-	D10
O001	-0.070(4)	0.005(4)	0
O002	-0.061(3)	0.007(3)	0
N003	0	0	-0.020(6)
C004	0	0	-0.040(8)
C005	-0.063(8)	-0.024(8)	-0.032(9)
C006	0	0	-0.012(9)
H00A	0	0	0.100(12)
H00B	0	0	0.104(12)
H00C	0	0	0.095(10)
H005	0	0	0.116(11)
H00D	0	0	0.107(12)
H00E	0	0	0.111(11)
H00F	0	0	0.115(13)

Table S4 Quadrupole parameters of L-alanine at 40K.

	Q20	Q21+	Q21-	Q22+	Q22-
O001	-0.026(4)	0	0	-0.040(4)	0.011(4)
O002	-0.040(4)	0	0	-0.027(4)	0.008(4)
N003	-0.048(6)	0	0	0	0
C004	0.055(7)	0	0	0.220(7)	0
C005	0.074(6)	0.026(7)	0.025(7)	-0.075(7)	0.004(6)
C006	-0.026(7)	0	0	0	0
H00A	0.070(13)	0	0	0	0
H00B	0.123(15)	0	0	0	0
H00C	0.088(15)	0	0	0	0
H005	0.065(14)	0	0	0	0
H00D	0.041(16)	0	0	0	0
H00E	0.045(15)	0	0	0	0
H00F	0.061(15)	0	0	0	0

Table S5 Octupole parameters of L-alanine at 40K.

	O30	O31+	O31-	O32+	O32-	O33+	O33-
O001	0	-0.041(5)	0.004(5)	0	0	0.048(5)	0.006(5)
O002	0	-0.023(5)	-0.012(6)	0	0	0.054(4)	-0.004(5)
N003	0.187(8)	0	0	0	0	0.145(7)	0
C004	0.269(10)	0	0	-0.176(9)	0	0	0
C005	-0.033(8)	-0.130(9)	-0.184(9)	0.023(10)	-0.010(7)	0.145(8)	-0.047(7)
C006	0.202(10)	0	0	0	0	0.159(9)	0

Table S6 Hexadecapole parameters of L-alanine at 40K.

	H40	H41+	H41-	H42+	H42-	H43+	H43-	H44+	H44-
O001	0.018(7)	0	0	-0.001(6)	-0.001(6)	0	0	-0.001(6)	0.003(5)
O002	0.007(7)	0	0	-0.009(6)	0.010(6)	0	0	0.028(5)	0.007(5)
N003	0.041(9)	0	0	0	0	-0.082(8)	0	0	0
C004	0.066(12)	0	0	-0.008(12)	0	0	0	-0.003(10)	0
C005	0.054(10)	-0.019(10)	-0.014(10)	-0.040(11)	0.053(10)	0.007(10)	-0.015(10)	0.037(9)	0.045(9)
C006	0.059(12)	0	0	0	0	-0.079(11)	0	0	0

Table S7 C_{jkl} of L-alanine.

	C111	C222	C333	C112	C122	C113	C133	C223	C233	C123
O001	0	0	0	0.000073(14)	0.000052(13)	0.000033(7)	0	0.000052(7)	-0.000019(4)	-0.00025(5)
O002	0	0	0	0	0	0	0	-0.000101(7)	0.000013(3)	-0.00023(5)
N003	0	0	0	-0.000057(14)	0	0	0	-0.000031(7)	0	-0.00023(5)
C004	0	0	0	0	0	0	0	0	0	-0.00024(5)
C005	0	0	0	0	0	0	0	0	0	-0.00024(5)
C006	0	0	0	0	0	0	0	-0.000064(10)	-0.000030(5)	-0.00024(5)

Table S8 D_{jklm} of L-alanine at 40K

	D_1111	D_2222	D_3333	D_1112	D_1222	D_1113	D_1333	D_2223	D_2333	D_1122	D_1133	D_2233	D_1123	D_1223	D_1233
O001	0.00023(2)	0	0.0000129(12)	0.000023(9)	0	0	0	0	0	0	0.0000180(17)	0.0000103(16)	0	0	0
O002	0.00039(3)	0	0.0000099(11)	0	0	0	0	0	0	0.000048(7)	0.0000216(18)	0.0000076(15)	0	0	0
N003	0.00023(2)	0.00018(2)	0.0000140(12)	0	0	0	0	0	0	0.000048(7)	0.0000228(17)	0.0000189(16)	0	0	0
C004	0.00032(3)	0.00016(2)	0.0000184(13)	0	0	0	0	-0.000021(5)	0	0.000059(7)	0.0000270(19)	0.0000203(17)	0	0	0
C005	0.00028(3)	0.00020(2)	0.0000198(13)	0	0	0	0	0	0	0.000056(8)	0.0000269(19)	0.0000237(18)	0	0	0
C006	0.00027(4)	0.00016(3)	0.000017(2)	0	0	0	0	0	0	0.000071(9)	0.000021(3)	0.000020(2)	0	0	0

Table S9 Refined MM parameters for taurine

Atom	Monopole	Dipole	Quadrupole	Octupole	Hexadecapole
S001	M1	D0	Q0	O0, O3+	H0, H3+
O002	M1	D1+, D1-	Q0, Q2+, Q2-	O1+, O1-, O3+, O3-	H0, H2+, H2-, H4+, H4-
O003	M1	D1+, D1-	Q0, Q2+, Q2-	O1+, O1-, O3+, O3-	H0, H2+, H2-, H4+, H4-
O004	M1	D1+, D1-	Q0, Q2+, Q2-	O1+, O1-, O3+, O3-	H0, H2+, H2-, H4+, H4-
N005	M1	D0	Q0	O0, O3+	H0, H3+
C006	M1	D1+, D1-	Q0, Q2+, Q2-	O1+, O1-, O3+, O3-	H0, H2+, H2-, H4+, H4-
C007	M1	D1+, D1-	Q0, Q2+, Q2-	O1+, O1-, O3+, O3-	H0, H2+, H2-, H4+, H4-
H00A	M1	D0			
H00B	M1	D0			
H00C	M1	D0			
H00D	M1	D0			
H00E	M1	D0			
H00F	M1	D0			
H00G	M1	D0			

Table S10 Dipole Parameters of MM for taurine at 85K with $\sin\theta/\lambda_{max}$=1.3736 Å^{-1} resolution.

	D11+	D11-	D10
S001	0	0	-0.022(15)
O002	-0.006(5)	-0.015(5)	0
O003	-0.037(6)	-0.008(6)	0
O004	-0.037(6)	-0.013(6)	0
N005	0	0	-0.024(8)
C006	-0.050(11)	-0.069(12)	0
C007	0.069(11)	-0.112(13)	0
H00A	0	0	0.062(15)
H00B	0	0	-0.019(14)
H00C	0	0	-0.013(16)
H00D	0	0	-0.136(14)
H00E	0	0	-0.049(15)
H00F	0	0	-0.105(14)
H00G	0	0	-0.161(13)

Table S11 Quadrupole parameters of MM for taurine at 85K with $\sin\theta/\lambda_{max}$=1.3736 Å^{-1} resolution.

	Q20	Q21+	Q21-	Q22+	Q22-
S001	-0.046(11)	0	0	0	0
O002	0.020(6)	0	0	-0.023(6)	0.024(6)
O003	0.034(7)	0	0	-0.034(6)	-0.013(6)
O004	0.028(6)	0	0	-0.029(7)	-0.006(6)
N005	-0.036(8)	0	0	0	0
C006	0.067(11)	0	0	0.015(9)	0.066(9)
C007	0.074(10)	0	0	-0.012(9)	0.028(9)

Table S12 Octupole parameters of MM for taurine at 85K with sinθ/λ_{max}=1.3736 Å^{-1} resolution.

	O30	O31+	O31-	O32+	O32-	O33+	O33-
S001	0.273(11)	0	0	0	0	0.329(11)	0
O002	0	0.008(8)	-0.003(7)	0	0	0.007(7)	0.001(7)
O003	0	-0.018(8)	0.022(8)	0	0	0.027(8)	0.019(7)
O004	0	-0.013(7)	0.029(7)	0	0	0.025(8)	0.008(8)
N005	0.131(11)	0	0	0	0	0.099(11)	0
C006	0	-0.158(13)	-0.174(14)	0	0	0.190(12)	-0.059(11)
C007	0	-0.128(12)	-0.217(14)	0	0	0.169(12)	-0.019(11)

Table S13 Hexadecapole parameters of MM for taurine at 85K with sinθ/λ_{max}=1.3736 Å^{-1} resolution.

	H40	H41+	H41-	H42+	H42-	H43+	H43-	H44+	H44-
S001	0.106(13)	0	0	0	0	-0.121(10)	0	0	0
O002	-0.011(11)	0	0	0.012(10)	0.004(9)	0	0	-0.002(9)	0.007(9)
O003	-0.011(11)	0	0	0.009(10)	0.008(10)	0	0	0.004(9)	0.001(9)
O004	-0.005(11)	0	0	0.000(10)	0.008(9)	0	0	-0.008(10)	0.019(10)
N005	-0.005(12)	0	0	0	0	0.009(12)	0	0	0
C006	0.057(16)	0	0	0.013(15)	0.050(16)	0	0	0.033(13)	0.029(14)
C007	0.043(16)	0	0	-0.054(15)	0.048(15)	0	0	0.020(14)	0.003(13)
